# Cytoreductive surgery in advanced epithelial ovarian cancer: a real-world analysis guided by clinical variables, homologous recombination, and BRCA status

**DOI:** 10.1016/j.ijgc.2025.101809

**Published:** 2025-04-04

**Authors:** Eliya Shachar, Yael Raz, Gilat Rotkop, Uriel Katz, Ido Laskov, Nadav Michan, Dan Grisaru, Ido Wolf, Tamar Safra

**Affiliations:** a Division of Oncology, Tel Aviv Sourasky Medical Center, Tel Aviv, Israel; b Faculty of Medical & Health Sciences, Tel Aviv University, Tel Aviv, Israel; c Department of Obstetrics & Gynecology, University of California, Los Angeles, CA, USA; d Department of Obstetrics & Gynecology, Tel Aviv Sourasky Medical Center, Tel Aviv, Israel; e Geha Mental Health Center, Petah Tiqva, Israel

**Keywords:** Ovarian Cancer, Interval Debulking, Primary Debulking, Cytoreduction, BRCA, Overall Survival, Homologous Recombination Deficiency

## Abstract

**Objectives:**

Guidelines endorse both interval and primary debulking cytoreductive surgeries in the treatment of epithelial ovarian cancer, emphasizing that the treatment strategy should be tailored to the patient’s clinical condition and tumor burden. Despite these recommendations, experts have yet to agree on a definitive surgical approach.

**Methods:**

A retrospective longitudinal analysis of 929 women diagnosed with advanced-stage (International Federation of Gynecology and Obstetrics stage III-IV) epithelial ovarian cancer between January 2002 and January 2025 was conducted. The effects of interval debulking surgery versus primary debulking surgery on median overall survival and progression-free survival were evaluated. Additionally, we aimed to identify patients who may benefit from a particular surgical approach based on clinical variables, mutation in either of the BRCA1 or BRCA2 genes, and homologous recombination profile.

**Results:**

A total of 929 patients were diagnosed with stage III to IV disease (87.2%) and underwent either primary debulking (*n* = 389, 41.9%) or interval debulking surgery following neoadjuvant chemotherapy (*n* = 540, 58.1%). Patients treated with primary debulking had a longer median overall survival than those treated with interval debulking surgery (68.40 months, 95% CI 62.92 to 76.45 vs 52.01 months, 95% CI 47.15 to 57.86, HR 1.2, *p* = .0004). However, when adjusted for age at diagnosis, stage, histology, BRCA status, and tumor resectability, multivariate analysis demonstrated no significant difference in survival between the two surgical groups (HR 1.15, 95% CI 0.96 to 1.39, *p* = .12). Younger women (<69 years), stage III, and BRCA-wild-type and/or homologous recombination proficient had longer survival with primary debulking than with interval debulking surgery (74.55 months, 95% CI 65.35 to 93.27 vs 55.98 months, 95% CI 48.10 to 64.79, HR 1.38, *p* = .03). Patients with a pathogenic BRCA variant or homologous recombination deficient profile had similar survival outcomes with either debulking approach, regardless of age and disease stage (*p* > .05). Propensity score analysis demonstrated comparable median overall survival with the two surgical timings (64.39 months, 95% CI 58.38 to 71.23 vs 57.69 months, 95% CI 50.66 to 64.79, HR 1.33, *p* = .27).

**Conclusions:**

Our findings support the use of neoadjuvant chemotherapy followed by interval debulking surgery without compromising survival outcomes, regardless of age and stage, particularly among harder-to-treat patients. We identified a specific subset of patients who may benefit from primary debulking surgery as the optimal intervention. These findings advocate for a personalized treatment approach and the potential for tailored surgical strategies guided by patient clinical variables, homologous recombination, and genetic factors.

## INTRODUCTION

Primary debulking surgery, followed by platinum-based chemotherapy, has long been the standard of care for advanced ovarian cancer. The primary objectives of primary debulking surgery are to accurately stage the disease and achieve optimal cytoreduction, ideally leaving no visible residual disease (R0 resection). Achieving R0 resection is strongly correlated with improved survival outcomes.^1,2^ Patients who are not candidates for optimal primary debulking due to extensive tumor burden or poor performance status may receive neoadjuvant chemotherapy followed by interval debulking surgery as an alternative approach.

The European Organization for Research and Treatment of Cancer (EORTC) 55971 trial demonstrated that neoadjuvant chemotherapy followed by interval debulking surgery is non-inferior to primary debulking surgery and adjuvant chemotherapy in terms of overall survival and progression-free survival, a finding corroborated by the CHORUS trial.^3-5^ These studies also indicated that interval debulking surgery is associated with lower perioperative morbidity and mortality than primary debulking surgery. Additional evidence from the SCORPION trial, along with retrospective studies by Bian and Glover and colleagues^6-8^ further reinforced these findings. The smaller JCOG0602 trial did not confirm the survival non-inferiority of neoadjuvant chemotherapy and interval debulking compared to primary debulking surgery.^9^ Instead, it showed variability in the efficacy of each treatment approach among different patient sub-groups.

The comparable overall survival and reduced morbidity associated with neoadjuvant chemotherapy and interval debulking surgery have led some gynecological oncologists, particularly in Europe, to advocate for interval debulking surgery as the preferred treatment for patients with advanced epithelial ovarian cancer.^3^ The European Society for Medical Oncology guidelines endorse both treatment approaches, emphasizing that the choice should be tailored to the patient’s clinical condition and tumor burden.^10^ Some experts argue that primary debulking should remain the preferred treatment for fit patients with advanced, resectable disease, while interval debulking is more suitable for those with poorer performance and nutritional status, and who are at higher risk for post-operative complications.^11^ The principle determinant in selecting the treatment approach is guided by the anticipated ability to achieve R0 resection while also minimizing morbidity.^12^ Moreover, advances in pre-operative imaging, such as positron emission tomography-computed tomography (PET-CT), have enhanced pre-treatment staging accuracy by informing the debulking approach.^13,14^ Laparoscopic assessment has become increasingly important in guiding surgical planning, with tools such as the Predictive Index Value, developed by Fagotti and colleagues,^15^ offering valuable insights into the extent of intraperitoneal disease.

It is well established that epithelial ovarian cancer with homologous recombination deficiency (HRD), particularly in the presence of BRCA mutations, exhibits biologically and clinically distinct prognostic characteristics that may help guide surgical management.^16^ BRCA1 and BRCA2 are tumor suppressor genes that play critical roles in maintaining genomic integrity. They encode proteins involved in the repair of double-stranded DNA breaks through homologous recombination, a key mechanism for DNA repair.^17^ Studies have demonstrated that both germline and somatic BRCA mutation status can predict the likelihood of achieving complete cytoreduction with primary debulking surgery and aid in treatment decision-making at diagnosis.^18,19^ However, the literature describing the impact of molecular testing on the surgical management of epithelial ovarian cancer is sparse.^16^ In this study, we retrospectively evaluated the influence of different debulking strategies on patient survival outcomes and aimed to identify whether a specific surgical approach is better suited for certain populations based on clinical features, HRD profile, and BRCA mutation status.

## METHODS

### Study Design, Setting, and Population

We conducted a retrospective longitudinal analysis of 1065 medical records of patients diagnosed and treated for epithelial ovarian cancer at Tel Aviv Sourasky Medical Center, Israel, from January 2002 to January 2025. The study was approved by the Institutional Ethics Committee (approval number: 0346-20-TLV).

Medical charts of women over 18 years of age who were treated with either interval or primary debulking surgery for advanced epithelial ovarian cancer (International Federation of Gynecology and Obstetrics stage III-IV) with various histologies, including serous-papillary, endometrioid, and other sub-types such as clear cell, carcinosarcoma, and mucinous ovarian cancer, were included in the study. Records of patients with non-epithelial histologies, borderline tumors, and low-grade serous carcinomas were excluded from the study. Additionally, patients with stage I and II disease were excluded, as they typically undergo upfront surgery and generally have a better prognosis.

### Surgery

The pre-operative workup included imaging (CT, magnetic resonance imaging, or PET-CT as indicated) to assess disease extent and resectability, histopathological confirmation of malignancy via biopsy or laparoscopy, evaluation of patient performance status using the Eastern Cooperative Oncology Group (ECOG) or Karnofsky scale, and laboratory tests, including the tumor marker CA125, liver and renal function tests, and hematologic parameters.

The decision between primary or interval debulking surgery was guided by a multidisciplinary tumor board comprising gynecologic oncology surgical specialists, medical oncologists, radiation oncologists, pathologists, and radiologists. The multidisciplinary tumor board team conducted a comprehensive review of imaging studies and pathohistological findings to determine an optimal treatment plan. Institutional guidelines consistent with international standards were followed to determine patient eligibility for primary debulking surgery. The inclusion criteria were advanced-stage epithelial ovarian cancer with anticipated complete resection based on imaging and multidisciplinary assessment, adequate performance status, and no contraindications to surgery. The exclusion criteria included extensive disease not amenable to optimal cytoreduction, poor performance status or severe comorbidities precluding surgery, and cases in which neoadjuvant chemotherapy was deemed necessary to improve surgical outcomes.

The surgeries were performed by board-certified specialists in gynecologic oncology with extensive experience in ovarian cancer surgery. The surgical team consisted of gynecological oncology specialists and, if indicated, hepatobiliary and gastrointestinal surgical specialists, reflecting the standard multidisciplinary care model at our center.

### Chemotherapy

All patients received carboplatin and paclitaxel chemotherapy, either as neoadjuvant chemotherapy prior to interval debulking surgery or following primary debulking surgery. The choice of chemotherapy scheduling regimen (paclitaxel-carboplatin 3-weekly or weekly treatment) was determined by the treating team, including the medical oncologist and gynecologic oncology surgeon, based on clinical parameters such as the patient’s performance status, comorbidities, and renal, hepatic, and hematologic function.^20^ Joint decision-making with the patient also played a role, considering factors such as treatment-related toxicity, quality of life, and the patient’s proximity to the treatment center. Generally, patients with poorer performance status, significant comorbidities, or a preference for minimizing toxicity to maintain their quality of life were more likely to receive weekly paclitaxel and carboplatin chemotherapy. In contrast, patients with a good performance status (ECOG 0-1) and minimal comorbidities typically receive the standard 3-weekly chemotherapy regimen.

### Genetic and Genomic Evaluation

The genetic evaluation comprised germline BRCA testing. Genomic instability scoring was performed to identify HRD. Patients who completed HRD testing were those who tested negative for the common germline pathogenic BRCA variants. Consequently, HRD patients include those with homologous recombination defects, including somatic BRCA mutations. The Israeli Ministry of Health first reimbursed this test in 2021.

### Research Variables and Outcome Measures

Patient data extracted from medical records included the following variables: demographics, past medical history, age, stage of disease at diagnosis, dates of diagnosis and surgery, residual tumor following cytoreductive surgery (complete resection [R0], residual tumor ≤1 cm (R1), residual tumor >1 cm [R2]), germline pathogenic variants in BRCA, HRD profile, CA125 tumor marker at diagnosis, and following 4 and 6 cycles of chemotherapy, administration of maintenance therapy with poly ADP-ribose polymerase (PARP) inhibitor and bevacizumab, and chemotherapy scheduling regimen (weekly or three-weekly carboplatin and paclitaxel).

Survival outcomes, including progression-free survival and overall survival, were evaluated across distinct patient sub-populations. Progression-free survival was calculated from treatment to either progression, death, or the last known follow-up. Overall survival was calculated from the date of diagnosis to either death or the last known follow-up. Patients were classified by genetic germline pathogenic variants in BRCA1 or BRCA2 and HRD profiles: HR proficient (HRP) or HRD.

### Statistical Analysis

Statistical analyses were performed using R version 4.4.1 (R Development Core Team, Vienna, Austria). Continuous variables were summarized as median and range and compared between groups using the *t* test. Categorical variables were summarized as numbers and frequencies (%) and compared using the χ^2^ test. Survival functions were demonstrated using the Kaplan-Meier method, and the effect of each group was assessed using the logrank test. Cox regression was used to predict the median progression-free survival and overall survival. The results are presented as HR with 95% CI. Two-tailed *p*-values <.05 was defined as statistically significant.

To address the inherent variability in a large longitudinal cohort treated over a wide time interval, we accounted for temporal trends in advances in surgical and adjuvant treatment by conducting multivariate analyses of patients diagnosed before and after 2018. This cutoff was chosen because systemic treatments, such as PARP inhibitors and bevacizumab, were available and approved by the Israeli Ministry of Health after 2017.

We also conducted a separate analysis of women diagnosed before December 2018 who survived for more than 7 years, ensuring a minimum follow-up period of 7 years to reduce bias and characterize long-term survivors treated with a particular debulking strategy.

Propensity score matching (PSM) was performed to minimize selection bias between patients treated with interval and primary debulking surgery. The propensity score was calculated using a logistic regression model adjusted for age, stage, histology, comorbidities, BRCA, HRD profile, PARP inhibitor, and previous malignancy as covariates. The Python (version 3.12) PsmPy package was used for matching the propensity score, with a caliper size of 0.2.^21^ Cohen’s d effect size was used to demonstrate an achieved balance between the treatment groups (Table S1). The covariates were checked for balance between the intention-to-treat arms, ensuring that there was no significant difference. A stratified Cox proportional hazards regression was conducted on the matched groups to assess the association between the surgical approach and overall survival while accounting for the matched pair structure. In accordance with the journal’s guidelines, we will provide our data for independent analysis by a selected team by the editorial team for the purposes of additional data analysis or for the reproducibility of this study in other centers, if requested.

## RESULTS

Among 1,065 women diagnosed with epithelial ovarian cancer between January 2002 and January 2025, 929 patients were diagnosed with stage III to IV disease (87.2%) and underwent either primary debulking surgery (389/929, 41.9%) or interval debulking surgery following neoadjuvant chemotherapy (540/929, 58.1%; Table 1). Patients treated with primary debulking surgery were significantly younger than those treated with interval debulking surgery (63.6 years, range; 26-91 vs 59.4 years, range; 28-88, *p* < .0001). Within the primary and interval debulking surgery groups, 36 (9.2%) and 135 (25.0%) patients were treated for stage IV disease (*p* < .0001).

A greater proportion of women with medical comorbidities (hypertension, diabetes, and hypercholesterolemia) were referred for interval debulking surgery than for primary debulking surgery (30.0% [*n* = 162] vs 20.3% [*n* = 79], *p* = .001). No statistically significant between-group differences were noted in the histological sub-types or BRCA status (*p* = .05 and *p* = .13, respectively). Among the 251 patients (27%) who underwent HRD testing, 105 (41.8%) were classified as HRD, 129 (51.4%) as HRP, and 17 (6.8%) had inconclusive results.

The rates of complete tumor resection (R0) were similar between the two surgical timings, with 65.4% (*n* = 248) in the primary debulking surgery group and 61.4% (*n* = 315) in the interval debulking surgery group (*p* = .24). A higher proportion of patients treated with primary debulking surgery received the three-weekly carboplatin-paclitaxel regimen compared to patients referred for interval debulking surgery (61.8% [*n* = 222] vs 51.5% [*n* = 266], *p* = .003). A greater proportion of patients treated with primary debulking surgery had platinum-sensitive disease than those treated with neoadjuvant chemotherapy followed by interval debulking surgery (82.3% [*n* = 306] vs 72.7% [*n* = 367], *p* = .001). A similar proportion of patients in both groups were treated with PARP inhibitors or bevacizumab (*p* = .06 and *p* = .33, respectively).

**Figure 1:**
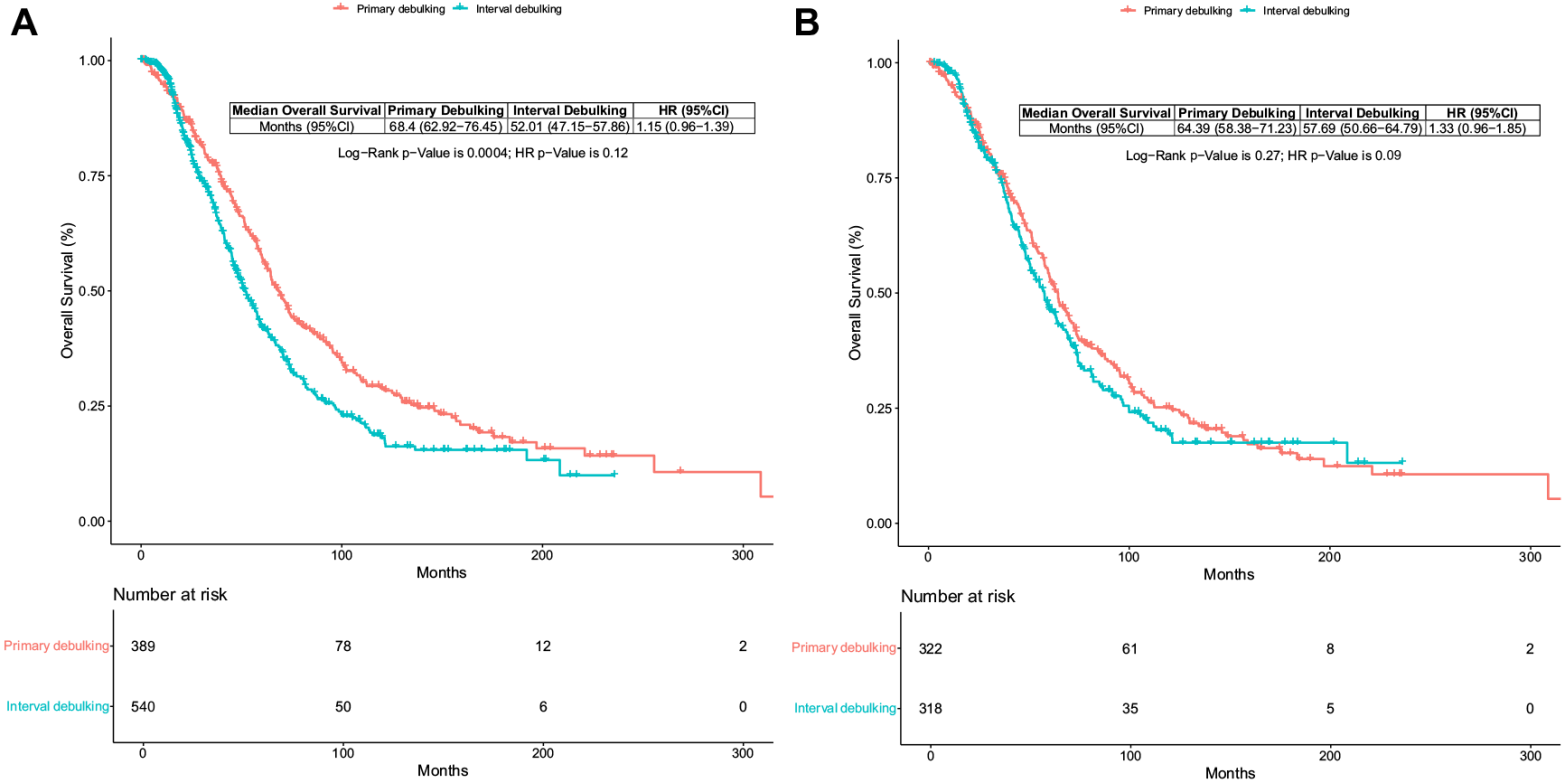
**A.** Kaplan-Meier survival curve of patients with advanced epithelial ovarian cancer stage III to IV disease treated with primary or interval debulking surgery. **B.** Propensity score matched Kaplan-Meier survival curve of patient cohorts with advanced epithelial ovarian cancer stage III to IV disease treated with primary or interval debulking surgery.

Patients treated with primary debulking surgery had a longer median overall survival than those treated with interval debulking surgery (68.40 months, 95% CI 62.92 to 76.45 vs 52.01 months, 95% CI 47.15 to 57.86, *p* = .0004). However, when adjusted for age at diagnosis, stage, histology, BRCA status, and tumor resectability, multivariate analysis demonstrated no significant difference in overall survival between the two surgical strategies (HR 1.15, 95% CI 0.96 to 1.39, *p* = .12; Fig. 1A). Age, stage, BRCA status, and resection rate were all significant factors for survivorship (*p* < .001).

To mitigate potential temporal bias with the introduction of newer therapies, a multivariate regression analysis was conducted to account for the timing of patients’ diagnosis and treatment before or after 2018. The analysis showed that the overall survival of patients treated with interval or primary debulking surgery was not significantly influenced by the year of treatment (HR 0.86, 95% CI 0.72 to 1.04, *p* = .12).

To address the potential bias of a retrospective study, we performed PSM between patients who underwent primary debulking surgery (*n* = 322) and interval debulking surgery (*n* = 318), matched for age at diagnosis, disease stage, histology, medical comorbidities, BRCA mutation status, HRD profile, PARP-inhibitor administration, and history of previous malignancy.

**Figure 2:**
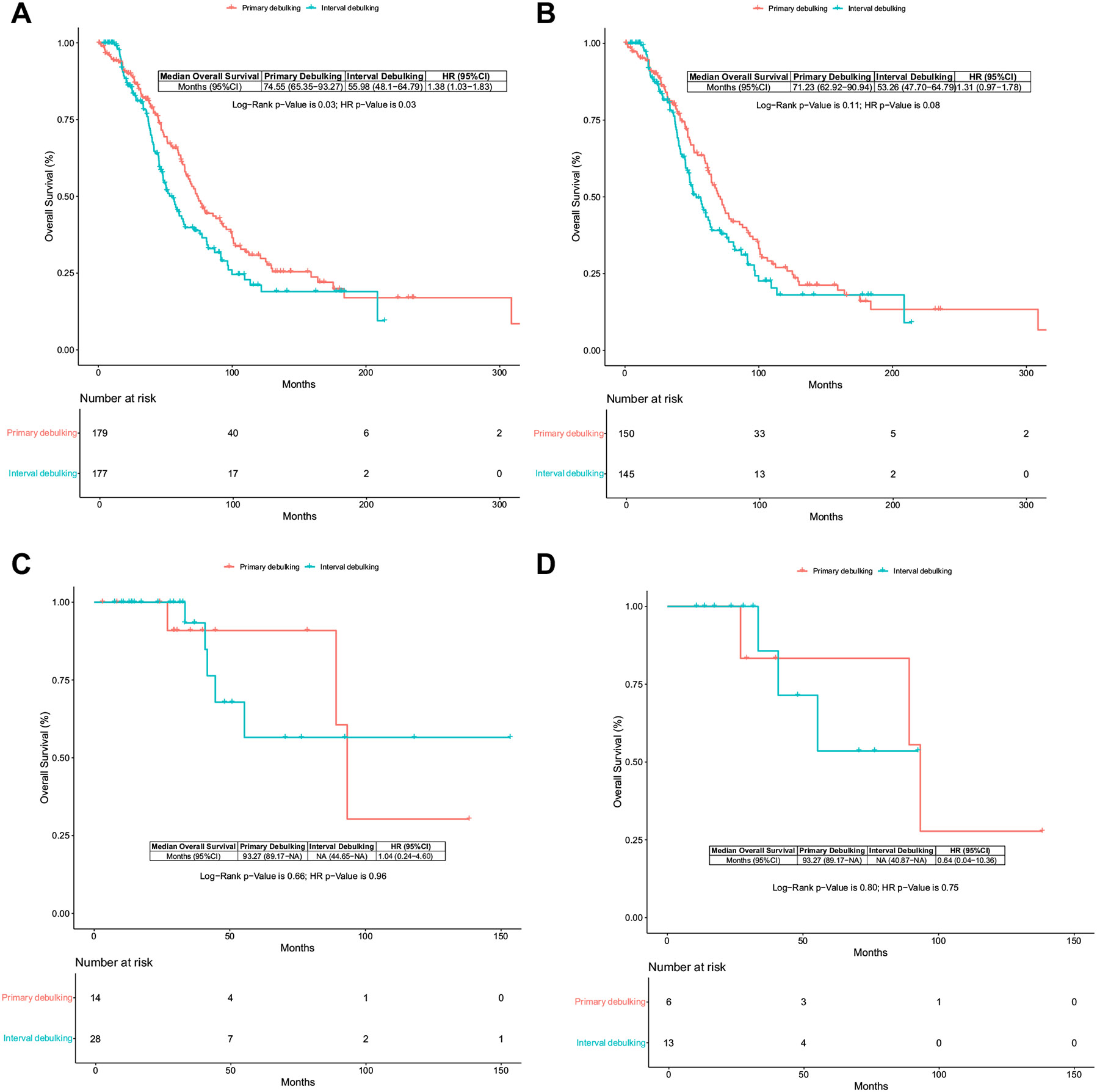
Kaplan-Meier curves. Survival curves of patients with advanced epithelial ovarian cancer sub-groups stratified by age, stage, and BRCA status and/or HRD profile. **A.** Overall Survival of young (<69 years old) patients with advanced epithelial ovarian cancer stage III disease BRCA-wild-type and/or HRP population treated with primary or interval debulking surgery. **B.** Propensity-score matched Kaplan-Meier survival curve of young (<69 years old) patient cohorts with advanced epithelial ovarian cancer stage III disease, BRCA-wild-type, and/or HRP profile treated with primary or interval debulking surgery. **C.** Overall survival of patients with advanced epithelial ovarian cancer stage III to IV, HRD profile, and germline BRCA Wild-type status treated with primary or interval debulking surgery. **D.** Propensity score matched Kaplan-Meier survival curve of patients with advanced epithelial ovarian cancer stage III to IV with HRD profile and germline BRCA Wild-type status treated with primary or interval debulking surgery. HRD, homologous recombination deficiency; HRP, homologous recombination proficient; NA, not available.

Across the matched groups, the median overall survival was numerically higher in the primary debulking surgery group than in the interval debulking surgery group, but this difference was not statistically significant (64.39 months, 95% CI 58.38 to 71.23] vs 57.69 months, 95% CI 50.66 to 64.79, *p* = .27; Fig. 1B). Multivariate analysis also demonstrated that median overall survival was not significantly different between the matched groups (HR 1.3, 95% CI 0.96 to 1.85, *p* = .09). Older age was significantly associated with shorter overall survival (HR 1.1, 95% CI 1.0 to 1.1, *p* = .007). Patients with BRCA mutations had significantly longer overall survival (HR 0.255, 95% CI 0.1 to 0.6, *p* = .0005) than those without a BRCA mutation. Sub-optimal resection with residual disease (R1/R2) was a strong predictor of shorter overall survival (HR 1.8, 95% CI 1.1 to 2.8, *p* = .0185). Stage, histology, and year of diagnosis were not statistically significant predictors of overall survival. Sub-group analysis of the matched groups based on age, stage, BRCA, and HRD did not demonstrate a survival benefit for a particular surgical approach (Fig. 2B).

Patients treated with primary debulking surgery had a longer median progression-free survival than those treated with interval debulking surgery (17.5 months, 95% CI 15.5 to 20.0 vs 12.7 months, 95% CI 11.3 to 15.1; HR 1.2, *p* = 0.005). When adjusted for age at diagnosis, stage, and BRCA status, multivariate analysis demonstrated a trend toward a difference in progression-free survival between the two surgical approaches (HR 1.15, 95% CI 0.98 to 1.34, *p* = .09). Age, stage, and BRCA status were all significant for progression-free survival (*p* < .05). To address temporal bias with the introduction of newer therapies, multivariate regression analysis was conducted to account for the timing of patients’ treatment before or after 2018. Progression-free survival of patients treated with interval or primary debulking surgery was not influenced by the treatment year (HR 0.99, 95% CI 0.85 to 1.15, *p* = .87).

To minimize bias in evaluating long-term outcomes (*N* = 666), we analyzed a cohort of patients with advanced epithelial ovarian cancer (stage III-IV) diagnosed before December 2018, with a follow-up period of over 7 years. Both univariate and multivariate logistic regression analyses were performed. Among these patients, 320 (48.8%) were treated with primary debulking surgery, and 336 (51.2%) were treated with interval debulking surgery.

In the univariate analysis, age at diagnosis, stage, resection rate, BRCA status, PARP-inhibitor administration, and surgical approach were significantly different between the two groups (Table S2). The multivariate model demonstrated that age at diagnosis, stage, sub-optimal/optimal debulking status, BRCA mutation status, PARP-inhibitor use, and surgical approach remained prognostic of survival. Bevacizumab treatment did not significantly affect survival in the multivariate analysis. Neoadjuvant chemotherapy followed by interval debulking surgery was associated with extended survival beyond 7 years (OR 0.63, *p* = .03).

Younger women (<69 years) with stage III disease, BRCA-wild-type (BRCA-WT), and/or HRP had a longer median overall survival with primary debulking surgery than those who received neoadjuvant followed by interval debulking surgery (74.55 months, 95% CI 65.35 to 93.27 vs 55.98, 95% CI 48.10 to 64.79, *p* = .03), with an HR of 1.38, 95% CI 1.03 to 1.83, *p* = .03 (Fig. 2A). The cytoreductive approach did not affect the survival of patients harboring a germline BRCA pathogenic variant, HRD molecular profile, or HRD with BRCA-WT germline testing, regardless of age or stage. Figure 2C and 2D demonstrate the subgroup of EOC patients with stage III/IV HRD profile and germline BRCA Wild-type status with comparable survival with primary and interval debulking surgery. Similar overall survival rates between primary and interval debulking surgery were also observed in older patients (>69 years) who were BRCA-WT or HRP, regardless of stage. The propensity score-matched sub-groups exhibited similar survival outcomes with either cytoreductive approach (Fig. 2B).

Although pre-treatment CA125 levels were significantly higher in patients who underwent interval debulking surgery than in those who underwent primary debulking surgery, the change in CA125 levels over time did not differ significantly between the groups after completing chemotherapy (Table S3). This suggests that the initially elevated CA125 levels observed in the interval debulking surgery group normalized to a similar range (<35 U/mL) as in the primary debulking surgery group following treatment.

## DISCUSSION

### Summary of Main Results

We evaluated the survival outcomes of patients with epithelial ovarian cancer who underwent neoadjuvant chemotherapy followed by interval debulking surgery compared to those who underwent primary debulking surgery to better understand the impact of surgical strategy on prognosis. Additionally, we considered the contribution of HRD and BRCA status to outcomes related to the chemotherapy-surgical sequence.

A significant proportion of patients underwent interval debulking surgery rather than primary debulking surgery, reflecting the increasing preference for neoadjuvant chemotherapy followed by interval debulking surgery in managing advanced disease, particularly in higher-risk patients with poor pre-treatment conditions. Our findings revealed no statistically significant difference in median overall survival between the two surgical timings when adjusting for age, stage, sub-optimal/optimal resection, and germline BRCA status through multivariate analysis and PSM. Patients undergoing interval debulking surgery had poorer characteristics; they were older, had more advanced stages, higher baseline CA125 levels, and greater comorbidities. Moreover, this cohort had a higher proportion of platinum-resistant diseases and a shorter median progression-free survival. Despite these unfavorable features, a comparable proportion of patients who underwent interval debulking surgery achieved complete tumor resection (R0), which is a critical prognostic measure and surrogate for overall survival. Notably, younger women (<69 years) with stage III disease, BRCA-WT, and/or HRP status derived the greatest survival benefit from primary debulking surgery compared to older women. In contrast, patients with germline BRCA mutations and/or HRD exhibited similar survival outcomes with either surgical approach, irrespective of age and stage. However, the propensity score-matched cohorts demonstrated comparable survival outcomes with either surgical timing strategy across sub-groups.

### Results in the Context of Published Literature

Our findings align with those of prior randomized trials, including EORTC 55971 and CHORUS, which demonstrated that neoadjuvant chemotherapy followed by interval debulking surgery results in similar survival rates compared to primary debulking surgery. The current parameters guiding advanced disease treatment include stage, disease burden, and patient performance status. A meta-analysis of the EORTC 55971 and CHORUS trials suggested that primary and interval debulking surgeries have varying efficacy based on disease extent: neoadjuvant and delayed cytoreductive surgeries improve outcomes in stage IV disease, while primary debulking surgery leads to longer overall survival in patients with abdominal disease <5 cm. Our study expands on this knowledge by incorporating patients’ clinical factors, genetic profiles, and genomic instability to refine surgical timing treatment sequencing.

In an unmatched analysis, neoadjuvant chemotherapy followed by interval debulking surgery appeared less effective, likely due to selection bias favoring neoadjuvant chemotherapy in patients with more advanced disease or poorer performance status. By balancing key factors such as stage, age, BRCA status, PARP-inhibitor administration, and HRD profile, the benefit of neoadjuvant chemotherapy and interval debulking surgery was evident, highlighting how unmatched retrospective studies may obscure treatment effects. Propensity score-matched analysis showed comparable survival between the two surgical timings after adjusting for confounders, a finding consistent across all subgroups. Our findings suggest that interval debulking can be confidently integrated into the treatment paradigm, particularly for patients with poor pre-treatment conditions who benefit from neoadjuvant therapy. Notably, interval debulking did not compromise the outcomes in patients with favorable clinical features.

The elevated pre-treatment CA125 levels in the interval debulking surgery cohort likely reflect a worse initial disease burden than that in the primary debulking surgery group. After four cycles of chemotherapy, the interval debulking surgery cohort maintained a higher median CA125 level, and this difference persisted after six cycles. By the sixth cycle, the median CA125 levels in both groups returned to the normal range. However, the rate of decline, measured by the slope of the linear regression, did not differ significantly between the cohorts. Despite worse baseline markers, patients who underwent interval debulking surgery ultimately responded to treatment. While useful, CA125 levels should be interpreted alongside other clinical parameters for a comprehensive prognostic assessment.

A multivariate sub-group analysis of younger patients with stage III disease, BRCA-WT and/or HRP molecular profiles demonstrated the greatest benefit of primary debulking surgery.

This observation may be attributed to a surgically amenable disease characterized by a genetic and homologous recombination profile that is relatively less responsive to systemic therapy. They represent a more challenging-to-treat population than patients harboring a germline pathogenic BRCA variant and/or HRD. These findings are supported by the literature,^16^ suggesting that primary optimal cytoreduction is a critical initial treatment in the sequencing of therapy and controlling disease burden.

In contrast, patients with BRCA mutations and/or HRD status demonstrate greater sensitivity to platinum-based chemotherapy and benefit from PARP-inhibitor maintenance therapy. These differences raise important questions regarding the ideal sequencing of systemic and surgical management, particularly when considering genetic and genomic instability status.^16,22,23^ The unique biological features of HRD tumors, particularly BRCA-associated epithelial ovarian cancer, likely contribute to treatment outcomes. BRCA-mutant tumors exhibit characteristic patterns of spread, including a tendency for peritoneal metastases with an expansive invasive pattern, “pushing metastasis,” rather than infiltrative spread.^18,24,25^ Distinct clinical and biological observations suggest that BRCA mutations may influence surgical cytoreduction.

The literature on patients with HRD in the absence of BRCA1/2 mutations is limited. Our findings support the expansion of indications for neoadjuvant chemotherapy and interval debulking surgery in advanced epithelial ovarian cancer with HRD and BRCA-WT, regardless of age and advanced stage.

Additionally, our study showed similar survival in patients who were BRCA-mutant, HRD, and in older patients regardless of BRCA status and homologous recombination profile.

However, there are concerns about the widespread adoption of neoadjuvant therapy and delayed debulking, as experimental data suggest that neoadjuvant chemotherapy and prolonged systemic treatments could lead to the selection of resistant clones in BRCA-proficient tumors or the development of secondary mutations restoring BRCA function.^26,27^ Prolonged neoadjuvant chemotherapy may lead to a desmoplastic reaction, reducing drug penetration into fibrotic tissues. These biological mechanisms could, in turn, promote the development of secondary resistance to platinum-based chemotherapy and PARP inhibitors.^16^

Contrary to our findings, the results from the sub-group analysis of the SOLO-01 study in BRCA-mutant patients treated with maintenance olaparib indicated that patients undergoing primary debulking surgery have a better prognosis than those receiving neoadjuvant and interval debulking surgery, suggesting that PARP-inhibitor treatment may not fully compensate for treatment sequencing and disease extent at diagnosis.^28^

The propensity score-matched analysis of our study population showed comparable survival outcomes with either surgical timing strategy across all sub-groups.

### Strengths and Weaknesses

One of the key strengths of this study is its large real-world cohort with long-term follow-up, which allowed for robust survival analyses. The use of PSM minimized selection bias, enhancing the comparability of the surgical groups. Additionally, the incorporation of genetic and HRD profiling provides a more personalized approach to decision-making regarding treatment-surgical sequencing.

However, this study had several limitations. This was a singlecenter, retrospective analysis, which may limit its generalizability to broader populations. The high prevalence of BRCA founder mutations in the studied cohort, reflective of the Ashkenazi Jewish population in Israel, may not be representative of the global ovarian cancer demographics. Studies have reported BRCA mutation rates of 34% to 48% among Ashkenazi Jewish women with ovarian cancer, far exceeding global figures.^29-32^ Furthermore, although we accounted for confounding factors, unmeasured biases inherent to retrospective studies cannot be entirely excluded. Finally, HRD testing has not been universally performed, limiting its application across all patients. To ensure broader applicability, future research should include diverse populations with varied genetic backgrounds, environmental exposures, and access to health care. Therefore, expanding the study cohort is crucial for validating and refining these findings.

## CONCLUSIONS

This study underscores the complexity of epithelial ovarian cancer treatment, highlighting the interplay between surgical approach and pre-treatment clinical conditions, such as age, disease stage, comorbidities, genetic status, and genomic instability profile. Our findings advocate for a more personalized treatment strategy, emphasizing the importance of genetic and HRD testing in guiding surgical decision-making. Notably, interval debulking surgery is a viable approach without compromising survival outcomes, particularly in harder-to-treat populations. These results challenge the traditional treatment paradigm and support a shift toward precision-based surgical strategies. Future prospective studies in diverse populations are essential to validate and expand these findings, ensuring broader applicability and improved patient outcomes.

## Supplementary Material

Supplementary

**Supplemental material** Supplementary data to this article can be found online at https://doi.org/10.1016/j.ijgc.2025.101809.

## Figures and Tables

**Table 1:** Demographic and Clinical Characteristics of the Study Population

Variables	Interval debulking *n* = 540	Primary debulking *n* = 389	*p*-Value
Diagnosis age, y, median (min, max)	63.6 (26, 91)	59.4 (28, 88)	<.0001
Stage			<.0001
Stage III	405 (75.0%)	353 (90.8%)	
Stage IV	135 (25.0%)	36 (9.2%)	
Histology			.05
Serous papillary	396 (73.3%)	269 (69.2%)	
Endometrioid/adenoma/poorly differentiated	136 (25.2%)	105 (27.0%)	
Other^a^	8 (1.5%)	15 (3.9%)	
BRCA status			.13
Unknown	125 (23.2%)	107 (27.5%)	
BRCA 1	86/415 (20.7%)	81/282 (28.7%)	
BRCA 2	42/415 (10.1%)	29/282 (10.3%)	
Negative	287/415 (69.2%)	172/282 (61.0%)	
Residual disease			.24
R0	315/513 (61.4%)	248/379 (65.4%)	
R1/R2	198/513 (38.6%)	131/379 (34.6%)	
Platinum sensitivity			.001
Platinum sensitive	367/505 (72.7%)	306/372 (82.3%)	
Platinum resistant	138/505 (27.3%)	66/372 (17.7%)	
Medical co-morbidities^b^			.001
Comorbidities	162 (30.0%)	79 (20.3%)	
No co-morbidities	378 (70.0%)	310 (79.7%)	
Carboplatin Paclitaxel Treatment regimen			.003
Weekly	251/517 (48.6%)	137/359 (38.2%)	
Three-weekly	266 (51.5%)	222/359 (61.8%)	
PARP inhibitor	52 (9.6%)	54 (13.9%)	.06
Bevacizumab	144 (26.7%)	92 (23.7%)	.33
Pretreatment CA125, units/mL	682 (5, 48,900)	311 (7, 20,000)	.0003
CA125 Slope	−114.29 (−4309.29, 40.86)	−40.72 (−2479.36, 41.11)	.16
Median overall Survival (mo)	52.01	68.40	.0004
Median progression-free survival (mo)	12.65	17.48	.005

Categorical variables are presented as numbers and percentages, and continuous variables are presented as medians (min, max). The denominator is indicated if it differs from the number of the entire patient group. Complete surgical cytoreduction with no macroscopic visual tumor, R1 and R2 were defined as macroscopic residual disease with a diameter of 0.1-1 cm (R1) or >1 cm (R2).

Abbreviations: max, maximum; min, minimum; PARP, poly ADP-ribose polymerase; R0, complete resection.

a Other histologies include mucinous, clear cell, and carcinosarcoma.

b Medical comorbidities include patients diagnosed with diabetes, hypertension, and hypercholesterolemia.

## WHAT IS ALREADY KNOWN ON THIS TOPIC

Guidelines endorse both interval and primary debulking cytoreductive surgeries for the treatment of advanced epithelial ovarian cancer, emphasizing that the treatment strategy should be tailored to the patient’s clinical condition and tumor burden.

## WHAT THIS STUDY ADDS

This study demonstrates that the choice of debulking approach in patients with epithelial ovarian cancer may be guided not only by tumor resectability but also by clinical factors, homologous recombination profile, and BRCA status. We identified sub-groups of patients with epithelial ovarian cancer for whom primary debulking surgery may be the preferred cytoreductive approach, including younger patients, those with stage III disease, BRCA-wild-type, and a homologous recombination proficient profile. In contrast, patients with a BRCA germline pathogenic variant or homologous recombination deficient profiles had similar overall survival outcomes regardless of surgical timing, independent of age and disease stage. Propensity score matching showed comparable survival outcomes between primary and interval debulking surgeries across all subgroups.

## HOW THIS MIGHT AFFECT RESEARCH, PRACTICE, OR POLICY

This study highlights the interplay between surgical strategies, clinical stage, genetic profile, and molecular genomic instability to tailor personalized surgical strategies. Future prospective studies are necessary to validate these findings in a broader and more diverse patient population.
