# Supplementary material for: Cytoreductive surgery in advanced epithelial ovarian cancer: a real-world analysis guided by clinical variables, homologous recombination, and BRCA status
Source: Int J Gynecol Cancer. Author manuscript; Available in PMC 2025 Jul 16. (PMC12265980; doi:10.1016/j.ijgc.2025.101809)
Supplement: Supplementary [file NIHMS2085224-supplement-Supplementary.docx]

**Supplementary**

**Table S1.** Propensity score matching demonstrating the effect size (calculating using Cohen’s D) small (d = 0.2), medium (d = 0.5), and large (d ≥ 0.8)

| **Variable matching** | **Effect Size before matching** | **Effect Size after matching** |
| --- | --- | --- |
| Age at diagnosis | 0.41 | 0.03 |
| Stage | 0.52 | 0.02 |
| Histology | 0.12 | 0.03 |
| Past malignancy | 0.04 | 0.07 |
| Comorbidities | 0.19 | 0.02 |
| BRCA | 0.22 | 0.03 |
| PARP inhibitor | 0.14 | 0.08 |
| Homologous recombination profile | 0.10 | 0.06 |

**Table S2**. **Logistic regression analysis of patients with advanced epithelial ovarian cancer treated with primary or interval debulking surgery and >7-Year Survival**

| **Variable** | **Univariate OR** | **p-value** | **Multivariate OR** | **p-value** |
| --- | --- | --- | --- | --- |
| Age at Diagnosis | 0.96 | <0.0001 | 0.98 | 0.01 |
| Stage III | 0.18 | <0.0001 | 0.28 | <0.0001 |
| Stage IV | 0.04 | <0.0001 | 0.11 | <0.0001 |
| Histology | 1.04 | 0.82 | 0.26 | <0.0001 |
| Debulking (R1/R2) | 0.20 | <0.0001 | 0.26 | <0.0001 |
| BRCA Mutation | 2.50 | <0.0001 | 1.85 | 0.007 |
| Avastin administration | 0.54 | 0.002 | 0.75 | 0.22 |
| PARP inhibitor administration | 2.72 | <0.0001 | 2.62 | 0.002 |
| Interval debulking surgery | 0.36 | <0.0001 | 0.63 | 0.03 |
|  |  |  |  |  |

Abbreviations-OR= odds ratio

**Table S3.** CA125 dynamics among advanced epithelial ovarian cancer patients treated with interval or primary debulking surgery

|  | **Interval debulking (N=328)** | **Primary debulking (N=227)** | **p-value** |
| --- | --- | --- | --- |
| CA125 pretreatment (median, IQR) | 688 (5-25,000) | 311 (7-20,000) | 0.0005 |
| CA125 after 4 cycles | 44 (1.6-3705) | 15 (1.9-2000) | 0.001 |
| CA 125 after 6 cycles | 15.8 (1.82-6000) | 11.6 (1.3-234) | 0.02 |
| CA125 Slope | -121.19(-4309-41.1) | -40.72(-2479.36-41.12) | 0.16 |
